# Engineering the Bone-Implant Interface

**DOI:** 10.1155/2018/4956491

**Published:** 2018-04-15

**Authors:** Francesco Mangano, Hassan Maghaireh, Josè Calvo-Guirado

**Affiliations:** ^1^University of Varese, Varese, Italy; ^2^University of Manchester, Manchester, UK; ^3^Universidad Católica de Murcia (UCAM), Murcia, Spain

The world of osseointegration has moved forward as a courtesy of the recent technological revolution which has presented itself in many forms, one of which is the introduction of new implant geometries and surfaces which is changing the surgical protocols, making everything easier, faster, and more predictable.

In fact, new implant macrotopographies have been introduced aiming to allow clinicians to obtain better and more predictable primary stability, therefore using dental implants as a solution to more challenging cases (such as areas of poor bone quality and/or in fresh postextraction sockets), with the possibility of anticipating nonconventional loading scenarios of the prosthetic superstructure, with the aim to provide positive patients outcome based on obtaining predictable short- and long-term results. Immediate placement of dental implants in fresh extraction sockets is usually highly appreciated protocol by patients, as it can provide satisfactory restorations at fraction of trauma, time, and cost. Similarly, the possibility of speeding up fitting the prosthetic superstructure is appreciated by patients, because it reduces treatment time and the related costs; in addition, it allows patients to avoid the discomfort and embarrassment of having to use provisional removable dentures during the healing period, with functional and aesthetic benefits.

In order to accelerate the bone healing processes, new implant micro- and nanotopographies have been introduced in the market. In fact, new surface treatments allow us to obtain micro- and nano-rough implant surfaces, characterized by a controlled micro- and nanotopography, able to geometrically stimulate and accelerate the bone healing processes: this can effectively enhance osseointegration, reducing healing times. These are important applications, in dentistry, of the tissue engineering concepts.

In the present thematic special issue, we have gathered several well conducted scientific and clinical papers that examine and describe the above concepts. This thematic special issue features a series of clinical studies on the effects of new implant macrotopographies on the implant stability, survival, and success in the short and long term. Moreover, we have collected a series of scientific articles (*in vitro *studies on cell cultures,* in vivo *animal histologic/histomorphometric studies,* in vivo *human histologic/histomorphometric studies, and clinical studies) on the effects of new macro-, micro-, and nanotopographies on osseointegration. Finally, we are delighted that this issue includes the first human histologic article in the dental literature, reporting on the 5-year results with an innovative strategy for enhancing aesthetics in implant treatment in the anterior maxilla utilising the root membrane/socket shield technique.

Only high quality research and clinical papers have been selected and published in the present thematic special issue. We really hope you will enjoy the reading and find it very interesting.

*Francesco Mangano* *Hassan Maghaireh* *Josè Calvo-Guirado*

